# Opposition to Youth e-Cigarette Prevention Campaigns on Twitter and TikTok: Cross-Platform Observational Mixed Methods Analysis

**DOI:** 10.2196/83791

**Published:** 2026-03-26

**Authors:** Chandler C Carter, Simon Page, Mateusz Borowiecki, Ganna Kostygina, Sherry L Emery, Miao Feng

**Affiliations:** 1 Social Data Collaboratory, Public Health Department, NORC at the University of Chicago, 55 E Monroe St 30th Floor, Chicago, IL, 60603, United States, 1 3127594000

**Keywords:** health campaigns, social media, tobacco industry, public health, health communication, e-cigarettes

## Abstract

**Background:**

Youth e-cigarette use rose sharply between 2013 and 2024 in the United States, prompting widespread prevention campaigns at national, state, and local levels. However, many campaigns encountered online opposition, sometimes leading to message distortion or campaign withdrawal. While previous studies have examined individual campaigns, little is known about how oppositional dynamics differ across social media platforms with distinct architectures.

**Objective:**

This study aimed to conduct a retrospective, cross-platform surveillance study of oppositional responses to US youth e-cigarette prevention campaigns, comparing tactics, themes, and engagement on Twitter (now X) and TikTok to inform platform-specific public health strategies.

**Methods:**

We collected Twitter (2014‐2020) and TikTok (2020‐2023) posts related to major US e-cigarette prevention campaigns using 15 campaign-specific hashtags and 4 verified prevention campaign handles. We included public, English-language posts from geographic regions allowed by the platforms. Machine learning classification and human coding were used to detect oppositional content, characterize narrative frames, and classify user types. Engagement was assessed using post-level metrics, including likes, shares, comments, and retweets. We analyzed message prevalence, engagement patterns, and oppositional themes.

**Results:**

On Twitter, opposition comprised 26.8% (83,074/310,207) of campaign-related posts overall but dominated certain campaigns (eg, *Still Blowing Smoke*: 6052/6113, 99%). A small cluster of advocacy and commercial accounts generated 57.8% of opposition retweets. Dominant narratives included questioning the credibility of health authorities, claims that prevention advertisements backfired, vaping rights, and product promotion. In contrast, TikTok opposition constituted only 3.5% (108/3127, 95% CI 3.1%-3.9%) of posts and was characterized by humor (71/108, 65.7%), mockery (48/108, 44.4%), and ironic portrayals of vaping (30/108, 27.8%). Individual creators comprised 76.1% (153/201) of accounts sharing prevention posts, and opposition videos used the visibility-boosting hashtag #fyp significantly more than prevention posts (51.9% vs 32.2%; *P*<.001). Despite inconsistent hashtag use, prevention posts achieved higher average engagement than oppositional content.

**Conclusions:**

This novel cross-platform, multicampaign analysis of opposition responses to e-cigarette prevention campaigns revealed how opposition reflects distinct platform architectures. Twitter opposition was highly coordinated and amplified by commercial and advocacy accounts, especially during regional campaigns. TikTok opposition was decentralized and humor-driven, aligning with the platform’s entertainment-oriented algorithm. The findings strengthen health communication by introducing a framework for evaluating platform-specific vulnerabilities and informing evidence-based campaign design. On Twitter, effective countermeasures may require real-time monitoring of social media discourse to support rapid responses to coordinated opposition. On TikTok, leveraging creator partnerships and remix-friendly content may help public health messages compete with entertainment-dominated discourse. Consistent hashtag use can strengthen engagement by minimizing the fragmentation of content visibility, and credible health sources should increasingly reinforce prevention narratives on both platforms. Greater platform accountability and transparency are needed to ensure that prevention content is not systematically deprioritized by algorithms relative to commercial promotion.

## Introduction

Following a long decline in youth tobacco use, the emergence of e-cigarettes in the 2010s prompted a resurgence in tobacco use among adolescents in the United States [1]. By 2023, approximately 10% of US high school students reported current e-cigarette use, making these devices the most popular tobacco product among youth [1]. Adolescents are particularly susceptible to nicotine addiction and may face additional respiratory, cardiovascular, and immunological harms [1,2]. This rise in youth use sparked significant public health concern, including concern about the potential for e-cigarettes to act as a “gateway” to other tobacco products. This was a key driver for the creation of federal and local prevention campaigns analyzed in this study [3-5]. Although interventions such as flavor bans have sought to curb youth appeal [6,7], e-cigarettes remain pervasive [8], underscoring the importance of effective prevention campaigns.

A diverse network of federal, state, and nonprofit stakeholders delivers tobacco prevention messages via television, print, and digital platforms [9-12]. These multichannel campaigns have demonstrated effectiveness in shaping risk perceptions and reducing initiation among youth [9,13-18]. However, the rise of social media presents new challenges for maintaining message integrity and evaluating broader impacts [19,20]. Despite generally being banned across traditional media, both direct and indirect tobacco marketing thrives on social media [21,22], with emerging evidence that such promotion is associated with tobacco use and susceptibility [23,24]. Furthermore, interactions among user behaviors, algorithmic feeds, and peer-to-peer interactions, such as remixing or hashtag hijacking, can amplify or undermine prevention efforts in ways not observed in traditional media environments [25-28]. These dynamics require a framework that accounts not only for content but also for how visibility, engagement, and narrative control are structured.

Simultaneously, counter-narratives thrive on social media [22,29,30]. Tactics such as “Twitter bombing” (ie, mass posting to overwhelm hashtags) or astroturfing (coordinated messaging disguised as organic support) allow commercial or advocacy interests to flood platforms with counter-narratives, creating misleading perceptions of widespread support or dissent [31-35]. Theory-based frameworks suggest that social media may allow commercial or advocacy actors to “set the agenda” in ways once restricted to institutional actors and traditional media. These frameworks also suggest that the structure of social media enables novel forms of gatekeeping that can amplify pro-vaping narratives, potentially increasing youth uptake while subverting prevention efforts [36-39]. Platform affordances shape these strategies: text-based environments such as Twitter (now X) facilitate rapid coordination and volume, while TikTok’s short-video format enables the spread of provaping content through easily digestible entertainment [28,40-42].

Drawing on theories of networked gatekeeping and principal-agent dynamics [37,38,43], we examine how digital platform architectures mediate the amplification of health messages versus counter-narratives. Networked gatekeeping theory highlights how control over content visibility is distributed across digital actors rather than centralized within traditional editorial institutions [37,43]. Principal-agent dynamics describe how misaligned incentives between message originators, such as public health agencies, and intermediaries, such as platforms or content creators, can shape how messages are disseminated and transformed [38]. In doing so, we explore how platform affordances shape engagement between public health campaigns and their challengers. Although previous research has examined antitobacco messaging or oppositional discourse on individual platforms, particularly Twitter and YouTube, little is known about how opposition dynamics differ across platforms with distinct sociotechnical architectures. Existing studies typically focus on single-platform case studies or specific campaigns [32], often without directly comparing how algorithmic design, user behaviors, and content norms shape oppositional engagement. This gap limits our understanding of how campaign messaging is received, reframed, or resisted in varied digital environments [31,32]. Our study is the first to provide cross-platform surveillance of opposition to US youth e-cigarette prevention campaigns. Specifically, we compare Twitter’s historical network dynamics (ie, retweet-based amplification by advocacy clusters) with TikTok’s contemporary algorithmic affordances (ie, individualized For You Page and entertainment-driven content formats). In our study, “opposition” is operationalized as content within specific campaign discursive spaces that diverges from or actively challenges official public health messaging. This approach examines patterns that may reflect platform-specific structural impacts of these divergent narratives, acknowledging the inherent complexity of online discourse without implying a simplistic binary. By analyzing both oppositional structures and engagement characteristics, we aim to illuminate platform-specific vulnerabilities and outline adaptive strategies for strengthening public health communication in increasingly participatory, algorithmically governed media ecosystems. We pursue three core aims: (1) to assess the volume and engagement of prevention campaign–related social media posts, (2) to identify the sources and tactics of oppositional content, and (3) to characterize platform-specific opposition dynamics to inform future strategy development.

## Methods

### Study Design and Rationale for Mixed Methods Approach

We conducted a US-based cross-platform surveillance study examining opposition to e-cigarette prevention campaigns on Twitter (2014‐2020) and TikTok (2020‐2023). We use the name “Twitter” throughout this study as our data collection period for this platform concluded in 2020, before its 2022 acquisition and rebranding as X. We analyzed posts related to campaigns at multiple levels of implementation, including national (eg, the Food and Drug Administration’s *The Real Cost*), state (eg, California’s *Still Blowing Smoke*), and local (eg, Chicago’s *Vaping Truth*). *The Real Cost* campaign initially launched in 2014 to curb cigarette use among youth, but by 2018, the campaign pivoted to address e-cigarette use [44].

This study used a convergent mixed methods design to integrate machine learning classification (quantitative) with thematic analysis (qualitative) to triangulate opposition patterns across platforms. Quantitative and qualitative findings were integrated at the interpretation stage by jointly examining how patterns in volume, engagement, and user type corresponded to emergent opposition themes across platforms. We selected this approach because quantitative classifiers alone cannot adequately capture multimodal content, while qualitative coding alone cannot scale across millions of posts.

Due to nonconcurrent time frames, we focused on identifying structural differences in oppositional engagement rather than conducting comparisons of post volume for cross-platform campaigns. Twitter hashtags were identified from campaign websites and through exploratory searches of social media content. Hashtags identified during Twitter data collection that also appeared on TikTok were used to collect data on TikTok, with TikTok data supplemented by searches for platform-specific hashtags (Table S1 in Multimedia Appendix 1).

This study is reported in accordance with the *American Psychological Association* Journal Article Reporting Standards for Mixed Methods Research (JARS–Mixed Methods) [45].

### Analytic Framework

#### Overview

Twitter and TikTok require distinct analytic approaches due to the text-based nature of the former and the multimodal nature (eg, video, audio, and text) of the latter. Machine learning techniques were only applied to Twitter, as text-based content is well suited for natural language processing techniques. In contrast, TikTok’s multimodal format presents challenges for automated classification, necessitating manual content and thematic coding to ensure accurate interpretation.

To systematically evaluate social media discourse related to e-cigarette prevention campaigns, we applied a 4-component framework assessing (1) message volume, (2) message content, (3) message sources, and (4) engagement metrics [40].

#### Message Volume

The number of tweets (Twitter) and videos (TikTok) was measured over time and by campaign. We used linear regression to assess changes in campaign-related post volume over time on each platform (from January 2020 to December 2023 on TikTok and from February 2016 to October 2020 on Twitter).

#### Message Content

To capture the advocacy stance (opposition vs prevention or neutral) of tweets, we used a supervised machine learning classifier. Opposition themes were identified using thematic analysis to explore the underlying opposition narratives. For TikTok, content analysis was used to systematically classify the advocacy stance of videos (opposition vs prevention vs neutral) and to identify high-level opposition themes. To further explore nuanced rhetorical strategies and narrative patterns within opposition posts, we conducted a thematic analysis.

#### Message Sources

For Twitter, user type was determined using a machine learning approach, while TikTok user classification was achieved through content analysis.

#### Engagement Metrics

For Twitter, engagement was operationalized as the number of retweets each post received. For TikTok, “interactions” comprised the sum of likes, comments, and shares per video, while views were analyzed separately as a measure of reach. TikTok-specific visibility-boosting features were also analyzed, including the proportion of videos using the hashtag #fyp as well as interactive video formats: duets (playing videos side by side from different users) and stitches (embedding clips from existing videos). Given these platform differences, engagement metrics were not compared directly across platforms. The validity of content analyses was assessed using Gwet’s AC1 statistic. The performance of the Twitter advocacy stance classification model was evaluated using *F*_1_-scores.

### Data Collection and Analysis

#### Twitter

We collected Twitter data using the now-deprecated Historical PowerTrack API, which provided enterprise-level access to the complete archive of public tweets matching defined rules on a monthly basis from January 2018 through April 2023. Our dataset includes all public English-language tweets (N=908,010) from February 2014 to October 2020 that matched 15 campaign-related hashtags associated with 14 distinct e-cigarette prevention campaigns, along with 4 verified campaign-affiliated accounts (Table S1 in Multimedia Appendix 1). Although the *Fresh Empire* campaign is focused on tobacco prevention, discussions of e-cigarettes appeared in posts using the campaign’s hashtag. Additionally, experimental evidence indicates that tobacco prevention messaging can increase perceived effectiveness for discouraging e-cigarette use, suggesting spillover effects across products [46,47].

Opposition content was identified using a supervised machine learning classifier trained on 2282 human-labeled tweets. Coders were instructed to label tweets as “oppositional” only when they expressed support for vaping, rejected the goals of prevention campaigns, or aligned with provaping advocacy. They also considered full textual context, including tone and source, to avoid misclassifying neutral or critical mentions of hashtags. Posts were classified as oppositional if they met any of the following criteria: (1) use of hashtags explicitly rejecting the prevention efforts (eg, #notblowingsmoke); (2) use of hashtags promoting vaping (eg, #vapingsavedmylife and #vapelife); (3) reference to vaping-related advocacy (eg, #wevapewevote); or (4) mention of organizations known for supporting vaping or harm reduction, such as the Consumer Advocates for Smoke-Free Alternatives Association. The classifier achieved a macro-averaged *F*_1_-score of 0.90 using 10-fold cross-validation, with a precision of 0.88 and recall of 0.92. An error analysis indicated that most misclassifications (false positives) occurred in cases where tweets mentioned health-related topics or campaign hashtags without clear oppositional framing.

#### TikTok

Data from 2020 to 2023 were collected in March 2025 in 2 phases using the TikTok Research API [48], an official platform-provided interface that allows approved researchers to access public content from designated geographic regions for research purposes. Our study focused on public content and restricted analysis to English-language posts, identified through metadata and manual review.

Phase 1 used 18 official hashtags and yielded 2563 posts. Phase 2 focused on 2 prominent national prevention campaign hashtags that were prominent in 2020: #ImmuneUpVapeDown and #ThisisQuitting, selected due to their prevalence and common spelling variations (eg, #thisisquitting was often misspelled as #thisisquiting; and #ImmuneUpVapesDown was also widely disseminated as #ImmuneUpVapeDown, a nonplural version of “Vape”). For *Immune Up*, both hashtag variations appeared in official promotional materials [49]. These 2 hashtags collectively generated over 2.1 million TikTok posts, from which we extracted as much content as application programming interface constraints allowed [50,51]. Hashtag queries were constructed using Boolean operators to include relevant variations and exclude unrelated campaigns.

To ensure our coded sample captured the full spectrum of content, from highly viral to more typical posts, we used quantile-stratified sampling based on total engagement (likes, comments, and shares combined). As engagement distributions on social media are heavily skewed, a simple random sample would risk underrepresenting the small number of high-impact posts that disproportionately shape discourse. We therefore ranked all 15,000 collected posts by engagement and divided them into 3 strata: the top 20% (high engagement), the middle 40% (moderate engagement), and the bottom 40% (low engagement). We then randomly sampled a proportionate number of posts from each stratum to generate our final coded sample of 800 posts. This approach ensured representation across visibility tiers and enhanced thematic diversity. All sampled posts were reviewed to exclude duplicates and nonsubstantive content. Across both phases, we collected 19,208 unique TikTok posts matching relevant prevention campaign hashtags. Of these, 15,000 (78.1%) posts with complete engagement metadata were eligible for ranking and quantile-stratified sampling. From this ranked set, we drew a proportionate stratified sample of 800 (5.3%) posts for manual coding. All posts from phase 1 of the data collection were manually labeled, resulting in a total of 3363 labeled posts. At the time of final analysis, 3127 (93.0%) labeled posts remained viewable and analyzable, with the remainder removed due to deletion and privacy restrictions. These removals accounted for approximately 7% of labeled TikTok posts and were treated as missing completely at random; no imputation was performed because missingness reflected post-level availability rather than content characteristics. A flow diagram summarizing the TikTok data collection and sampling workflow is provided in Figure S1 in Multimedia Appendix 1.

### Content and Thematic Coding

#### Twitter

To develop opposition content themes, we used a thematic analysis approach. Two coders reviewed the top (highest tweet volume) 1000 oppositional messages from the most active users. We focused on the most active users, as their high volume of content is more likely to shape the overall narrative [52]. The coders then collaboratively identified themes, which were refined and grouped together until we arrived at four principal themes: (1) criticism of and mistrust in public health institutions (eg, claims of health authorities spreading inaccurate information and critiques of regulations), (2) claims of a boomerang effect of prevention advertisements (eg, advertisements triggering a desire to use e-cigarettes), (3) vaping rights and advocacy (eg, movements such as #ivapeivote), and (4) product promotion (eg, deals and coupons).

#### TikTok

For TikTok’s short-form, video-first format, 5 trained coders developed and applied a structured codebook through an initial inductive coding phase, in which codes were generated directly from the data, followed by the integration of deductive codes informed by prior literature. The codebook included (1) user-level categories: account type (individual, organization, community [eg, fan accounts or news aggregators], and other), sector (health and government), and advocacy stance (protobacco or anticampaign and antitobacco or procampaign); and (2) video-level features: substance type (e-cigarette, other tobacco product, and other substance), advocacy stance, public service announcement framing (eg, conveying facts about substance harms or providing cessation resources), quit narratives (personal accounts of quitting a substance), brand mentions, product promotions, and entertainment or humor (overall; eg, skits, lip-syncing, jokes, and memes). Intercoder reliability was strong across all dimensions (Gwet AC1>0.81). To uncover more nuanced opposition narratives, we used the same thematic analysis approach applied to Twitter. Two coders jointly reviewed opposition posts (n=108), refining themes through iterative discussion, and identified six principal themes: (1) harm reduction or health claims (eg, mentions of any e-cigarette or tobacco-related health outcomes or comparative risk of tobacco smoking vs vaping), (2) personal testimony about vaping or tobacco benefits, (3) mockery of prevention campaigns (eg, using parody or satire), (4) dissatisfaction with advertisements, (5) ironic or hyperbolic references to vaping (eg, to comment on vaping culture or one’s own vaping behaviors, including decisions to ignore prevention information), and (6) claims of a boomerang effect of prevention advertisements. The harm reduction or health claims were each evaluated for accuracy based on information from the Food and Drug Administration’s Tobacco Education Resource Library [53]. Thematic saturation was reached when no new themes emerged during the final coding iterations.

### Twitter User Classification

Twitter user classification followed a 2-step process. First, 2 coders labeled a random sample of 300 accounts (with up to 10 tweets per account) into 4 mutually exclusive categories that were determined using a grounded theory approach: health (health-related organizations or individuals, including researchers and health care providers), commercial (organizations or brands explicitly selling or promoting tobacco products), provape advocacy (organizations or individuals advocating for pro–e-cigarette or tobacco policies or agendas), and organic (individuals with identity cues who are not public figures or influencers). Agreement was strong for the first 3 categories (AC1>0.80) and moderate for organic (AC1=0.72). Next, we used GPT-4o, accessed via an OpenAI instance on Microsoft Azure, to classify the same 300 accounts using structured prompts (Multimedia Appendix 2) and compared manual labels with GPT [54]. Accounts unrelated to the abovementioned categories or lacking sufficient information were classified as “other.” Compared to human labels, GPT-4o achieved *F*_1_-scores of 0.91 for organic and health accounts, 0.89 for advocacy accounts, and 0.96 for commercial accounts. On the basis of this validation, we applied the same prompting strategy to classify all unique users in the analytic data, using a unique user identifier available in the Twitter data. However, as Twitter display names were used as a feature for GPT’s user classification and are nonunique (eg, users can change their display names), we opted to classify unique users in the Twitter data, sorted by highest follower count: this ensured we classified unique users using the most popular observation per unique identifier, where popularity is determined by the follower count. Final classifications were cleaned and merged with post-level data to analyze account type by post stance (opposition vs prevention or neutral). These classifications were used to contextualize quantitative patterns of opposition volume and engagement and to inform the interpretation of qualitative opposition themes across platforms. All codebooks used for data labeling are available upon request.

### Ethical Considerations

All research activities for this study were approved by NORC at the University of Chicago’s Institutional Review Board (IRB 23061378) and determined to meet the criteria for exemption under Exempt Category 4, as defined in US federal regulations (45 CFR 46.104) for using existing, publicly available social media data. The social media data were collected in accordance with each platform’s terms of service and without direct interaction with users; therefore, informed consent was not required by the institutional review board. Privacy and confidentiality were safeguarded by ensuring that all data used in the analysis were publicly available and that all reported data were deidentified. No identifiable information or images of individual users are included in the manuscript or supplementary materials. Finally, no compensation was provided, as the study involved no direct interaction with or intervention upon human subjects.

## Results

### Campaign-Related Post Volume and Opposition Trends

We collected a total of 908,010 tweets referencing 14 e-cigarette prevention campaigns or mentioning the organization that produced the campaign from 2014 to 2020. After filtering for tweets explicitly referencing campaign names or hashtags, 310,207 (34.2%) tweets were retained for analysis. On TikTok (2020-2023), we analyzed 3127 viewable posts after excluding deleted, private, or non-English or non–US-based content.

**Figure 1.**
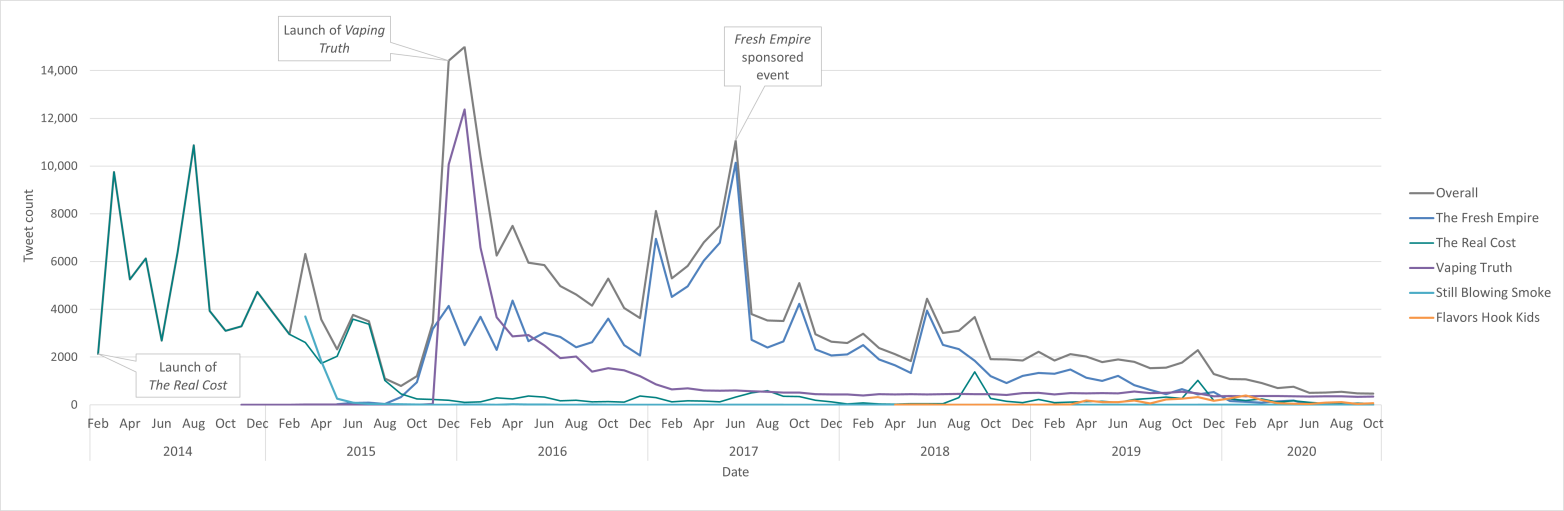
Campaign-related tweet counts over time, overall and by the 5 campaigns with the highest tweet count (2014-2020). This figure illustrates the overall volume of discourse and key peaks corresponding to campaign launches.Table 1

Twitter campaign activity peaked during major campaign launches: March 2014 (n=9742) and August 2014 (n=10,867), corresponding with the rollout of *The Real Cost*, and January 2016 (n=14,989), coinciding with the launch of *Vaping Truth* (Figure 1). A peak in June 2017 (n=11,059) aligned with a *Fresh Empire*–sponsored event. From February 2016 to the end of the study period (October 2020), tweet volume declined significantly (β=–122 tweets per month, 95% CI –144.08 to –99.17; *P*<.001). On TikTok, campaign-related content exhibited smaller peaks, with the largest in October 2020 (n=995) during the launch of *Immune Up Vapes Down*. Post volume declined modestly but significantly from January 2020 to December 2023 (β=–5 posts per month, 95% CI: –7.91 to –1.02; *P*=.01).

On Twitter, opposition content comprised 26.8% (83,074/310,207) of campaign-related posts, peaking in March 2015 (3766/6314, 59.7%) during the *Still Blowing Smoke* campaign and in January 2016 (11,821/14,989, 78.9%) during the *Vaping Truth* campaign. On TikTok, opposition was lower overall (108/3127, 3.5%) but rose modestly from 1.3% (27/2088) in 2020 to 5.6% (23/408) in 2023, peaking at 9.9% (28/282) of campaign-tagged posts in 2021, during *The Real Cost* campaign.

Opposition intensity varied by campaign and platform. On Twitter, among campaigns with more than 30 posts, state and local campaigns faced the most sustained counter-advocacy. Opposition comprised 99% (6052/6113) of content related to *Still Blowing Smoke*, 97.3% (70,300/72,270) of *Vaping Truth*–related content, and 27.3% (35/128) of messages about *The Vape Talk*. In contrast, national campaigns faced lower opposition: 3.4% (3125/93,246) for *The Real Cost* and 2.3% (3079/134,343) for *Fresh Empire* (Table 1). On TikTok, among campaigns with more than 30 posts, the highest opposition proportions were found in *The Real Cost* (56/246, 22.8%), *Vapes Down* (22/178, 12.4%), and *Ditch Juul* (4/42, 9.5%). Nine campaigns had limited TikTok presence (<30 posts).

**Table 1.** Proportion of campaign-related tweets classified as opposition by platform, overall and by campaign hashtag^a^.

Platforms and Campaigns	Opposition, n (%)	Total, n (%)
Twitter		
Total	83,074 (26.8)	310,207 (100)
StillBlowingSmoke	6052 (99)	6113 (2)
VapingTruth	70,300 (97.3)	72,270 (23.3)
TheVapeTalk	35 (27.3)	128 (0.0)
VapingEquals	18 (15.5)	116 (0.0)
FlavorsHookKids	383 (12.9)	2974 (1)
TheDirtyTruth	22 (10.5)	209 (0.1)
IJustDidn’tKnow	15 (9.7)	155 (0.0)
VapeFreeNovember	42 (7.1)	592 (0.2)
FactsOverFlavor	2 (6.4)	31 (0.0)
TheRealCost	3125 (3.4)	93,246 (30.1)
FreshEmpire	3079 (2.3)	134,343 (43.3)
TikTok		
Total	108 (3.5)	3127 (100)
TheRealCost	56 (22.8)	246 (7.9)
VapesDown	22 (12.4)	178 (5.7)
DitchJuul	4 (9.5)	42 (1.3)
DotheVapeTalk	2 (6.1)	33 (1.1)
EscapeTheVape	2 (5)	40 (1.3)
DitchVape	7 (3)	230 (7.4)
ImmuneUpVapesDown	11 (0.9)	1290 (41.3)
ThisIsQuitting	5 (0.5)	1011 (32.3)

a Total Twitter figures include all tweets collected using 15 prevention campaign-related hashtags associated with 14 distinct campaigns. Total TikTok figures reflect the subset of labeled posts with viewable videos at the time of analysis. Only campaigns with >30 posts are shown individually; others are included in the total.

### Engagement Patterns and Visibility-Boosting Features

On Twitter, retweets accounted for 59.7% (185,315/310,207) of campaign posts. Prevention tweets were retweeted 3.6 times more often than opposition posts. The most retweeted campaigns were *Still Blowing Smoke* (4977/6113, 81.4%) and *The Real Cost* (66,060/93,246, 70.8%). Opposition users also posted more frequently than prevention or neutral users (9.6 vs 1.6 posts per user). Engagement was highly concentrated: the top 10% of opposition accounts generated 84.4% (34,080/40,378) of opposition retweets, and the top 1% accounted for 63.9% (25,782/40,378).

On TikTok, opposition posts accounted for a smaller proportion of total posts (108/3127, 3.5%), compared to prevention posts (410/3127, 13.5%). Opposition posts also received lower average engagement (3103 vs 12,008 interactions (likes, comments, and shares). The median engagement was 40.0 (IQR 13.0‐209.8) for prevention posts and 44.5 (IQR 16.8‐128.0) for opposition posts. TikTok interactions (likes, comments, and shares) were driven by a small number of high-impact users: 97.9% (328,128/335,074) of opposition interactions came from just 11 accounts (the top 10%).

Opposition content used visibility-boosting platform features more frequently. The hashtag #fyp (“For Your Page”) appeared in 51.9% (56/108) of opposition posts, compared to 32.2% (132/410) of prevention posts. Use of interactive video formats, such as duets and stitches, was similar across conditions (opposition posts: 6/108, 5.6% vs prevention posts: 20/410, 4.9%).

### Opposition Themes

Prominent themes in opposition messaging varied by platform. On Twitter, key opposition themes included (1) criticism of and mistrust in public health institutions, (2) claims of a boomerang effect of prevention advertisements, (3) vaping rights and advocacy, and (4) product promotion. Many high-volume users aligned with advocacy movements such as #ivapeivote or #ABillionLives, framing vaping as a personal liberty or health-related right.

On TikTok, opposition content was primarily entertainment-oriented. The most frequently observed themes included general humor or entertainment (71/108, 65.7%), mockery of e-cigarette prevention campaigns (48/108, 44.4%), and ironic or hyperbolic references to vaping (30/108, 27.8%). One-quarter of opposition posts (27/108, 25%) visually depicted an e-cigarette. Expressions of dissatisfaction with prevention advertisements were present in 15.7% (17/108) of posts. Less frequently coded themes included claims of a boomerang effect of prevention advertisements (n=4, 3.7%), product promotion (n=2, 1.9%), personal testimony about the benefits of tobacco or vaping (n=2, 1.9%), and harm reduction or health claims (n=2, 1.9%). Notably, all health-related claims (n=3, 2.8%) contradicted established scientific consensus (eg, people with asthma are typically found to be healthier when they vape) [53].

### Content Creator and Source Analysis

On Twitter, among users with nonmissing profile descriptions, organic users constituted the majority (42,446/49,809, 85.2%), followed by other (6850/49,809, 13.8%), health (2264/49,809, 4.5%), provape advocate (1570/49,809, 3.2%), and commercial accounts (951/49,809, 1.9%). However, among opposition posters, provape advocacy (1376/5465, 25.2%) and commercial (724/5465, 13.2%) accounts were overrepresented, and organic users (2926/5465, 53.5%) were underrepresented compared to overall figures.

Among accounts posting prevention or neutral content, representation by health-related sources was 4.8% (2139/44,344).

On TikTok, individuals made up 91.8% (2023/2204) of labeled users, community accounts made up 3.1% (69/2204), organizations made up 2.8% (61/2204), and other types of accounts made up 2.3% (51/2204). A small proportion (29/2204, 1.3%) of users were categorized as health- or government-affiliated accounts.

Among opposition posters, individuals were the most prevalent (95/103, 92.2%), followed by community accounts (4/103, 3.9%), organizations (2/103, 1.9%), and other types of accounts (2/103, 1.9%). There was no representation by health or government sectors. Provape advocacy represented 6.8% (7/103) of opposition accounts.

Among accounts posting prevention messages, 76.1% (153/201) were individuals, 14.4% (29/201) were organizations, 5% (10/201) were community accounts, and 4.5% (9/201) were some other type of account. Health-related accounts constituted 10% (20/201) of these accounts, while 0.5% (1/201) were government related.

### Hashtag Variation and Engagement

Campaign engagement was shaped by both deliberate co-optation strategies and hashtag variation. Most prominently, opposition to *Still Blowing Smoke* on Twitter was marked by extensive use of the counter-hashtag #notblowingsmoke [32], which highlighted the claim that e-cigarette vapor is less dangerous than cigarette smoke and appeared alongside the official campaign hashtag #stillblowingsmoke in 53.7% (3248/6052) of the campaign’s opposition tweets.

On TikTok, engagement patterns were affected by the concurrent use of multiple similar hashtags associated with the same campaign. Across the study period, #ImmuneUpVapeDown was tagged in 3552 posts, while #ImmuneUpVapesDown appeared in 1184 posts. Although #ImmuneUpVapesDown appeared less frequently, posts using this hashtag generated higher average engagement, with 11,271 views and 1380 engagements per post, compared to 7831 views and 1105 engagements per post for #ImmuneUpVapeDown. This reflects a 1.4-fold increase in views and a 1.3-fold increase in engagements for #ImmuneUpVapesDown.

A similar pattern was observed for the *This is Quitting* campaign. The official hashtag, #thisisquitting, appeared in 13,114 posts, whereas the common misspelling, #thisisquiting, appeared in 434 posts. Despite its lower volume, posts with the misspelled hashtag generated slightly higher average engagement (1653 vs 1536 interactions per post). However, these posts received lower average viewership compared to posts with the official hashtag (10,877 vs 14,163 views per post).

## Discussion

### Principal Findings

Our study offers one of the first cross-platform examinations of digital opposition to youth e-cigarette use prevention campaigns, identifying distinct structures, engagement patterns, and thematic frames across Twitter and TikTok. We found that opposition was more prevalent and coordinated on Twitter, driven by commercial and advocacy accounts. In contrast, TikTok opposition was decentralized and humor-driven. Despite these differences, prevention content generally achieved higher engagement than oppositional posts on both platforms. These findings highlight the extent to which oppositional activity is shaped not only by campaign content but also by the unique affordances, user cultures, and algorithmic logics of each platform. Understanding these dynamics is essential. Responsive and resilient prevention strategies must be tailored to the evolving digital media landscape.

Opposition took divergent forms across platforms, reflecting structural and cultural asymmetries. On Twitter, opposition was highly coordinated and driven by commercial and advocacy accounts that leveraged hashtag hijacking and volume-based amplification. These patterns reflect classic principal-agent dynamics and networked gatekeeping. In both cases, vested interests influence which narratives gain visibility and shape policy discourse at scale. Organized advocacy networks and commercial promotion directly contested health communication and regulatory narratives. Their messages reflected direct challenges to health institutions, calls for deregulation, claims of a boomerang effect, harm reduction narratives, and product promotion.

In contrast, TikTok opposition was decentralized and creator-led, with individual users relying on algorithmically favored formats, such as humor, parody, and remix. Rather than mounting explicit policy critiques, TikTok users often reframed prevention campaigns as objects of satire or trivialization through “duets” that mock campaign messages, lip-syncing to songs about e-cigarette use, or comedic skits. Twitter’s text-centric environment and low production barriers facilitate more textual coordination, while TikTok’s video-first interface favors performative and affective engagement [55-57].

Prior research [32-35] has documented Twitter “bombing” or astroturfing and branding appropriation; our findings suggest that TikTok’s entertainment-driven opposition presents a structurally distinct challenge to campaign coherence. Importantly, public health institutional accounts comprised only 10% of all prevention-aligned TikTok content, indicating that the discourse is overwhelmingly dominated by individual creators. While this does not speak directly to impact or effectiveness, it underscores the relatively modest presence of institutional voices within platform-native discourse. The prevalence of mockery on TikTok, with the opposition’s significantly higher use of visibility-boosting hashtags like #fyp, aligns with an algorithmic logic that privileges engagement over informational accuracy. This makes evidence-based public health content less salient unless adapted to the platform’s affective and performative norms. The presence of both coordinated and decentralized opposition across platforms suggests that prevention campaigns face 2 qualitatively distinct credibility challenges: organized counter-messaging that seeks to influence policy and diffuse creator-driven content that may erode credibility through parody and cultural reframing.

Engagement dynamics further underscore the differences in the nature of opposition between platforms. On Twitter, opposition users posted or retweeted repeatedly, suggesting coordinated persistence. On TikTok, opposition was more episodic and reliant on the virality of individual posts. Although opposition content more frequently used visibility-boosting features such as #fyp, it did not consistently outperform prevention content in engagement, indicating that tagging alone does not guarantee amplification. These dynamics reflect how each platform’s algorithmic logic privileges particular forms of participation. Twitter rewards repeated real-time posting, trending topics, and follower networks, while TikTok prioritizes personalized content that aligns with user engagement patterns and preferred entertainment formats [55,56].

This pattern of engagement is further illustrated by the concentration of opposition interactions among a small number of high-impact TikTok users. Most opposition interactions (likes, comments, and shares) came from just 11 accounts representing the top 10% of opposition users. This aligns with TikTok’s influencer-driven model, where a small number of content creators can disproportionately shape discourse and visibility. For prevention campaigns, this suggests a strategic opportunity to increase engagement. This can be achieved by forming partnerships with credible, platform-native, youth-aligned influencers who can compete with oppositional voices in reach rather than relying on broad content distribution.

Notably, opposition visibility did not uniformly suppress engagement with prevention content, particularly on TikTok, suggesting that volume alone does not determine engagement and impact. This underscores the need for platform-specific intervention approaches. On Twitter, effective approaches should focus on detecting coordinated opposition and preemptively framing policy discourse to counter specific antiregulatory narratives. On TikTok, campaigns should invest in creative, platform-native formats, such as duets, remixes, or trending audio strategies, to maximize resonance with youth audiences while expanding public health visibility within the algorithmic ecosystem.

Future research should examine how competing narratives influence youth perceptions of e-cigarette use risk, assess the impact of unsupported and unauthorized health claims on public understanding, and explore regulatory approaches to moderating coordinated opposition. Further work is needed to examine how evolving algorithmic infrastructures govern the diffusion and visibility of public health content, including how oppositional discourse adapts in increasingly sophisticated ways. In addition, campaign-by-campaign thematic analyses could help disentangle the influence of specific prevention messages from broader platform-level dynamics and determine whether certain prevention themes predict opposition. These future efforts would also benefit from deeper integration of theory, particularly frameworks such as networked gatekeeping to better explain the structural vulnerabilities of public health messaging online.

Across both platforms, disruptive hashtag dynamics emerged in two forms: (1) strategic co-optation or hijacking (eg, #notblowingsmoke) and (2) hashtag fragmentation, which included both user-generated “drift” (ie, unintentional spelling variations such as #thisisquiting) and inconsistencies introduced by campaigns themselves (eg, simultaneous promotion of 2 similar but nonidentical hashtags for a single campaign). While hashtag drift can sometimes reflect authentic engagement and meme-like spread, inconsistent use of multiple official hashtags introduces risks. It can confuse the audience, dilute message visibility, and complicate evaluation. To address this, campaigns should promote a single, memorable, and unique hashtag consistently across all materials and partner networks. This helps minimize the hashtag-level fragmentation of campaign content visibility reflected in our findings and strengthens the integrity of message tracking.

Real-time monitoring can help detect and address hijacking and hashtag drift. Campaign staff should set up real-time alerts using social listening tools for sudden spikes in volume or shifts in sentiment. Such monitoring could signal a coordinated hijacking effort like the one seen with some counter-campaigns observed in regional campaigns. Quantitative monitoring should be paired with daily qualitative reviews of the content being posted under the hashtag to understand how the narrative is evolving.

Finally, prevention campaigns need preplanned rapid response protocols to counter oppositional messages. These should extend beyond reactive posts. There should be a preplanned communications protocol that may include a playbook of preapproved messages, coordinated content amplification, and activating credible messengers. Such practices are especially important in environments such as Twitter, where opposition is coordinated and can quickly flood the e-cigarette prevention discourse. It is also crucial in environments where algorithmic feeds (eg, TikTok’s For You Page) may amplify small differences in framing, format, or metadata, thereby influencing content visibility and diffusion in unpredictable ways.

### Limitations

This study has several limitations. First, platform-level constraints, particularly inconsistencies in the TikTok Research API, limited the volume of analyzable content, especially for high-volume campaigns. In addition, it remains unclear whether promoted or sponsored advertisement content is consistently included via the application programming interface; if excluded, our analysis may underrepresent campaign materials delivered through paid placements. Second, while observed differences in discourse across platforms could be attributed to structural or programmatic features of the platforms, they could also reflect variation in data collection periods or user demographics. As Twitter and TikTok data were collected from nonoverlapping periods (2014‐2020 and 2020‐2023, respectively), our goal was not to directly compare levels of opposition or engagement across time. Rather, each dataset reflects the dominant usage period of the platform for prevention campaigns, and our analysis focuses on platform-specific opposition dynamics within those distinct temporal contexts. Third, while engagement metrics provide useful indicators of content visibility and performance, they do not capture user intent, sentiment, or behavioral outcomes. Therefore, high engagement should not be equated with either campaign impact or message suppression. Additionally, Twitter user classification relied on mutable display names rather than persistent identifiers, which constrained our ability to characterize user behavior at scale. Finally, our analyses do not take into account secular trends in platform activity, such as the long-run decline in Twitter activity between 2014 and 2019. As our analysis is primarily concerned with proportions and themes of content, this limitation does not substantively impact our results [58].

### Conclusions

This study systematically compared oppositional dynamics across e-cigarette prevention campaigns on 2 major social media platforms using a mixed methods approach. Unlike previous studies that focused on singular platforms or campaigns, this study compared several e-cigarette prevention campaigns on Twitter and TikTok to reveal how distinct sociotechnical architectures shape opposition strategies. Campaign resilience in digital environments depends on more than message design alone. Our findings offer actionable guidance to strengthen digital health communication across platforms. Public health institutions must adapt to platform-specific architectures. On Twitter, strategies to counter opposition content should include real-time surveillance to enable rapid response to coordinated opposition. On TikTok, public health entities may improve engagement by investing in creator partnerships and using visibility-enhancing content formats. Sustained investment in creator collaboration is especially critical to counter entertainment-driven opposition narratives with content forms already familiar and appealing to youth audiences. Across both platforms, campaigns should ensure consistent branding to prevent potential audience fragmentation and message dilution from hashtag variations. Additionally, given the relatively small presence of credible health sources in the prevention discourse, they should be used more frequently to reinforce prevention narratives. On Twitter, credible health voices are essential for combating coordinated advocacy networks, while on TikTok, they should complement influencer partnerships and entertainment-oriented formats.

Meanwhile, regulatory agencies and health campaigns should also advocate platform accountability, including algorithmic transparency. This entails, at minimum, requiring platforms to disclose whether prevention content is systematically downranked compared to commercial promotion and to provide greater elevation of verified public health sources.

## Supplementary material

10.2196/83791 Multimedia Appendix 1 TikTok data collection and sampling workflow and overview of e-cigarette social media campaigns by hashtag, account handle, region, platform, and post count.

10.2196/83791 Multimedia Appendix 2 ChatGPT prompt for Twitter user classification.
