# Supplementary material for: Opposition to Youth e-Cigarette Prevention Campaigns on Twitter and TikTok: Cross-Platform Observational Mixed Methods Analysis
Source: J Med Internet Res. 2026 Mar 26;28:e83791. doi: 10.2196/83791 (PMC13021103; doi:10.2196/83791)
Supplement: Multimedia Appendix 1 [file jmir-v28-e83791-s001.pdf]

Figure S1. TikTok data collection and sampling workflow

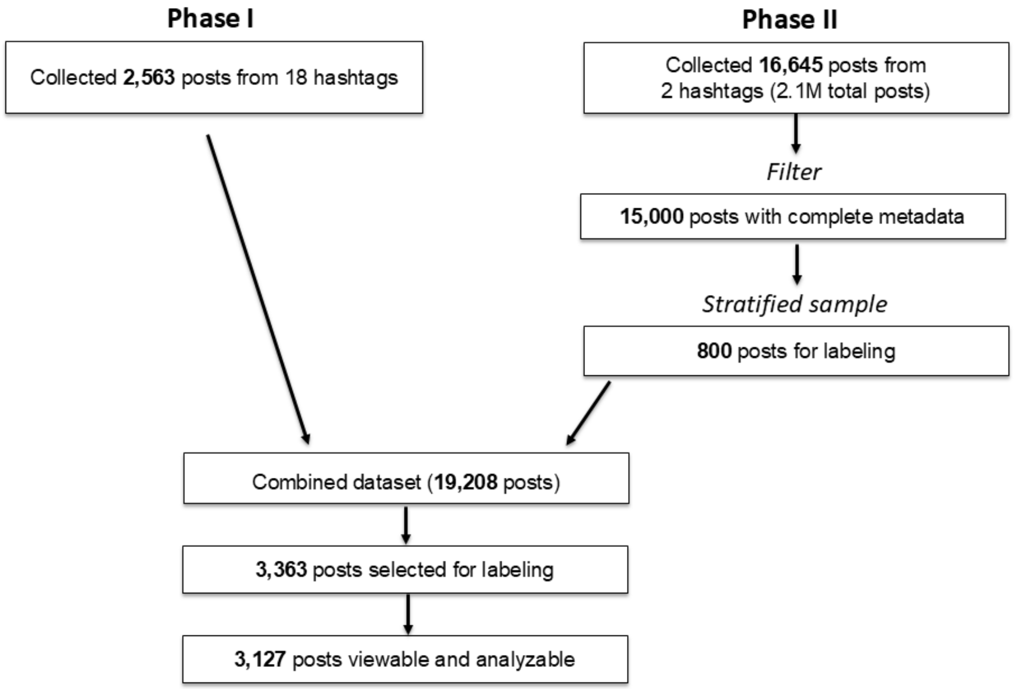

Table S1. Overview of e-cigarette social media campaigns by hashtag, account handle, region, platform, and post count

Hashtag(s)	Campaign Name	Region	Account/user (Twitter only)	Twitter	TikTok	Twitter post count	TikTok post count
#freshempire	Fresh Empire	National	@freshempire	X		134,343	
#therealcost	The Real Cost	National	@FDATobacco	X	X	93,246	271
#VapingTruth	Vaping Truth	Chicago, IL		X	X	72,270	2
#stillblowingsmoke	Still Blowing Smoke	California		X		6113	
#FlavorsHookKids	Flavors Hook Kids	California		X	X	2974	-
#VapeFreeNovember	Vape Free November	Colorado		X		592	
#TheDirtyTruth	The Dirty Truth	Delaware		X		209	
#IJustDidntKnow	I Just Didn't Know	Kentucky	@healthyky	X		155	
#thevapetalk (Twitter) and #dothevapetalk (TikTok)	The Vape Talk	Pennsylvania		X	X	128	33
#VapingEquals and #WeAreJCPS	Vaping Equals	Jefferson County	@JCPSKY	X		116	
#FactsOverFlavor	Facts Over Flavor	Arizona		X		31	
#BeSmartDon'tStart	Be Smart, Don't Start	Vermont		X	X	12	13
#BehindTheHaze	Behind the Haze	Kentucky		X	X	11	1
#TheRealDealOnVaping	The Real Deal on Vaping	Pima County		X		7	
#thisisquiting and #thisisquitting	This is Quitting	National			X		13,548
#thisisquitting*	"	"			X		13,114
#thisisquting	"	"			X		434
#immuneupvapesdown* and #immuneupvapedown	Immune Up Vapes Down	National			X		4736
#immuneupvapesdown*	"	"			X		1184
#immuneupvapedown	"	"			X		3552
#ditchvape	N/A	N/A			X		2441
#vapesdown	Vapes Down	Texas			X		247
#ditchjuul	Ditch Juul	National			X		52
#escapethevape	Escape the Vape	Minnesota			X		42
#quitnowindiana	Quit Now Indiana	Indiana			X		25
#vapesaretrash	Vapes Are Trash	National			X		19
#mylifemyquit	My Life My Quit	National			X		15
#endteenvaping	N/A	N/A			X		2
#betuffdontpuff	BeTuffDontPuff	South Dakota			X		1

Note: For hashtags with spelling variations, an asterisk (*) indicates the official campaign hashtag. For campaigns with multiple associated hashtags, individual hashtag counts may include overlapping posts.
